# Human Metapneumovirus Seasonality and Co-Circulation with Respiratory Syncytial Virus — United States, 2014**–**2024

**DOI:** 10.15585/mmwr.mm7411a1

**Published:** 2025-04-03

**Authors:** Ndey Bassin Jobe, Erica Rose, Amber K. Winn, Leah Goldstein, Zachary D. Schneider, Benjamin J. Silk

**Affiliations:** ^1^Epidemic Intelligence Service, CDC; ^2^Coronavirus and Other Respiratory Viruses Division, National Center for Immunization and Respiratory Diseases, CDC.

**Summary**

**What is already known about this topic?**

Human metapneumovirus (hMPV) and respiratory syncytial virus (RSV) are members of the *Pneumoviridae* family and cause similar illnesses. In the United States, RSV typically circulates from fall until spring, and hMPV typically circulates from winter through spring.

**What is added by this report?**

Circulation of both hMPV and RSV declined significantly during the 2020–21 respiratory virus season when the COVID-19 pandemic began. During the 2022–23 and 2023–24 seasons, RSV season offsets have been occurring earlier than usual in late winter, and typical hMPV circulation patterns have returned, with peak circulation in spring.

**What are the implications for public health practice?**

Understanding hMPV and RSV co-circulation patterns could guide timing and prioritization of clinician-directed testing, prompt detection of and response to outbreaks, and help ensure preparedness of health care systems for seasonal increases in respiratory viral illnesses.

## Abstract

Human metapneumovirus (hMPV) infections cause acute respiratory illness and lower respiratory tract disease. Respiratory syncytial virus (RSV) is a closely related virus within the *Pneumoviridae* family, and hMPV and RSV infections are associated with similar clinical manifestations. Although no specific antiviral therapies or vaccines exist for hMPV, vaccines and monoclonal antibody products are available to protect against severe RSV disease. This report summarizes hMPV circulation relative to the timing of RSV epidemics before, during, and after the COVID-19 pandemic. Polymerase chain reaction testing results reported to the National Respiratory and Enteric Virus Surveillance System during July 2014–June 2024, were analyzed. Before the COVID-19 pandemic, the median hMPV season onset, peak, and offset occurred in early January, late March, and early June, respectively (median duration = 21 weeks). The 2021–22 season was atypically long (35 weeks); seasonality reverted to more typical patterns during the 2022–23 and 2023–24 seasons. In the two COVID-19 pandemic seasons (2021–22 and 2022–23) and one postpandemic season (2023–24), RSV offsets occurred earlier in January (2021–22 and 2022–23) or March (2023–24) than before the pandemic, when the median offsets occurred in April. The annual interval from peak RSV to peak hMPV circulation increased from a prepandemic median of 11.5 weeks (range = 2–17 weeks) to 19 weeks (range = 19–20 weeks) during and after the pandemic. Fewer than 5 weeks of cocirculation of RSV and hMPV occurred in most regions during the 2022–23 and 2023–24 seasons. Real-time surveillance of RSV and hMPV co-circulation patterns can help guide clinician-directed testing and supportive care, optimize the use of prevention products, prompt detection of and response to outbreaks, and help ensure health care system preparedness for seasonal increases in illnesses.

## Introduction

Human metapneumovirus (hMPV) was first identified as a cause of respiratory illness in 2001. Children, older adults, and persons with compromised immune systems are at higher risk for hMPV-associated lower respiratory tract infections (bronchitis, bronchiolitis, and pneumonia) (*1*). hMPV can also exacerbate asthma and chronic obstructive pulmonary disease. Among adults in the United States, an estimated 12.1 hMPV-associated hospitalizations per 100,000 persons occurred each year during 2016–2019 (*2*). hMPV and respiratory syncytial virus (RSV) belong to the *Pneumoviridae* family* and cause similar respiratory illnesses (*3*). During 2008–2014, hMPV circulated from January to May in the United States (*4*). hMPV circulation has consistently followed seasonal RSV epidemics which, before the COVID-19 pandemic, typically occurred during October–April, with winter co-circulation of the viruses. However, the COVID-19 pandemic was associated with disruptions in hMPV and RSV seasonality: the 3% positivity threshold used to calculate the beginning and end of the season, and thus describe seasonality, was not met during the 2020–21 season (*5*). This report describes hMPV seasonal circulation patterns during July 2014–June 2024 and identifies changes in national and regional hMPV and RSV co-circulation during and after the pandemic. Understanding co-circulation patterns could help ensure that public health officials detect and respond to outbreaks promptly and help ensure that health care systems are prepared for seasonal increases in the incidence of viral respiratory illnesses.

## Methods

### Data Source

The National Respiratory and Enteric Virus Surveillance System (NREVSS)^†^ is a passive, laboratory-based surveillance system that monitors viruses circulating in the United States. Each week, participating clinical, commercial, and public health laboratories voluntarily report aggregate numbers of tests performed and positive detections to NREVSS by testing method (antigen detection, polymerase chain reaction [PCR] testing, or virus isolation).

Numbers of hMPV and RSV PCR tests and detections reported to NREVSS during July 2014–June 2024 were analyzed. A surveillance year was defined as July–June.^§^ Data from laboratories that consistently reported to the system (i.e., with an average of at least 10 PCR tests per week for ≥30 of 52 weeks of the surveillance year) were included in the analyses of each virus. The number of laboratories with consistent reporting of hMPV PCR test results to NREVSS increased from 62 in 34 states during the 2014–15 season to 122 in 38 states during the 2023–24 season. The number of laboratories with consistent reporting of RSV PCR test results increased from 77 in 36 states during the 2014–15 season to 239 in 44 states during the 2023–24 season.

### Analysis of Seasonality

For each virus, the weekly percentage of positive test results was calculated^¶^ to assess virus circulation, and smoothed trend data were visualized with 3-week (national) and 5-week (regional) centered moving averages. hMPV and RSV season onsets and offsets were defined as the first and last of ≥2 consecutive weeks with ≥3% positive test results, respectively, and the season peak was defined as the week with the highest percentage of positive test results. The median onset, peak, and offset for the prepandemic (2014–15 to 2019–20) seasons were compared with the two COVID-19 pandemic seasons (2021–22 and 2022–23) and one postpandemic season (2023–24). The change in hMPV circulation during the 2020–21 COVID-19 pandemic surveillance year was described, but the season was not analyzed because of the disruption of circulation (i.e., the 3% positivity threshold was not exceeded) (*5*). The number of weeks when RSV and hMPV seasons overlapped was estimated as each season’s interval before the RSV offset week (≥3% positive test results in the last of ≥2 consecutive weeks) and after hMPV onset week (≥3% positive results in the first of ≥2 consecutive weeks), nationally and by U.S. Department of Health and Human Services (HHS) Region.** All data analyses and visualizations were performed using R software (version 4.4.0; R Foundation). This activity was reviewed by CDC, deemed not research, and was conducted consistent with applicable federal law and CDC policy.^††^

## Results

### hMPV Circulation

The prepandemic six-season median of the median annual number of tests performed per reporting laboratory was 3,838.5; the three-season median number of tests performed per reporting laboratory during and after the pandemic increased to 4,634 (Table 1). Before the pandemic, a median of 476,169.5 hMPV tests were reported per surveillance year (median positive results = 3.2%). During and after the pandemic, the median number of hMPV tests reported increased by 92% to 914,660 per surveillance year (median positive results = 3.5%). The early COVID-19 pandemic was associated with a 98% decline in hMPV circulation, with 370 (0.07%) of 511,902 hMPV PCR test results reported as positive during the 2020–21 season (Figure).

**TABLE 1:** Numbers of reporting laboratories, human metapneumovirus polymerase chain reaction tests performed, and positive test results reported, by surveillance year, before, during, and after the COVID-19 pandemic — National Respiratory and Enteric Virus Surveillance System, United States, 2014–2024

Surveillance yr	No. of states reporting	No. of laboratories consistently reporting	No. of tests performed	Median no. of tests performed per laboratory (IQR)	No. of positive test results (%)	Minimum no. of weekly tests performed	Maximum no. of weekly tests performed	Minimum no. of weekly positive tests (%)	Maximum no. of weekly positive tests (%)
**Pre–COVID-19 pandemic**									
2014–2015	34	62	297,284	3,620 (3,640)	9,078 (3.1)	2,932	9,475	3 (0.1)	583 (8.4)
2015–2016	37	81	379,594	3,877 (3,609)	15,583 (4.1)	3,559	13,202	10 (0.2)	1,084 (9.2)
2016–2017	40	91	480,520	3,896 (4,461)	14,688 (3.1)	4,347	16,902	19 (0.3)	911 (7.6)
2017–2018	39	92	471,819	3,800 (4,643)	19,524 (4.1)	4,152	16,982	34 (0.6)	1,036 (8.1)
2018–2019	42	97	509,074	3,740 (5,402)	16,610 (3.3)	4,444	14,938	12 (0.2)	895 (6.2)
2019–2020	40	100	560,821	4,206 (5,259)	16,640 (3.0)	4,247	28,290	2 (<0.1)	1,966 (7.0)
**Six-season median**	39.5	91.5	476,169.5	3,838.5*	16,096.5 (3.2)^†^	4,199.5	15,920	11 (0.2)^†^	973.5 (7.8)^†^
**COVID-19 pandemic**									
2020–2021	35	109	511,902	3,185 (4,504)	370 (<0.1)	5,005	12,219	1 (<0.1)	37 (0.3)
2021–2022	40	121	897,316	4,479 (6,647)	31,806 (3.5)	12,221	28,216	34 (0.3)	1,795 (7.7)
2022–2023	39	118	914,660	4,634 (6,986)	32,038 (3.5)	10,620	28,436	72 (0.5)	1,927 (10.8)
**Post–COVID-19 pandemic** ^§^									
2023–2024^†^	38	122	926,652	4,674 (7,816)	23,889 (2.6)	10,763	25,274	31 (0.2)	1,472 (7.9)
**Three-season median^¶^**	39	121	914,660	4,634*	31,806 (3.5)^†^	10,763	28,216	34 (0.3)^†^	1,795 (7.9)*

* The median of the median number of tests performed annually per laboratory.

^†^ The median of the number, minimum, and maximum (percentage) of positive test results.

^§^ The COVID-19 pandemic public health emergency declaration ended May 2023.

^¶^ During and after the pandemic, the median includes the 2021–22, 2022–23, and 2023–24 seasons.

**FIGURE:**
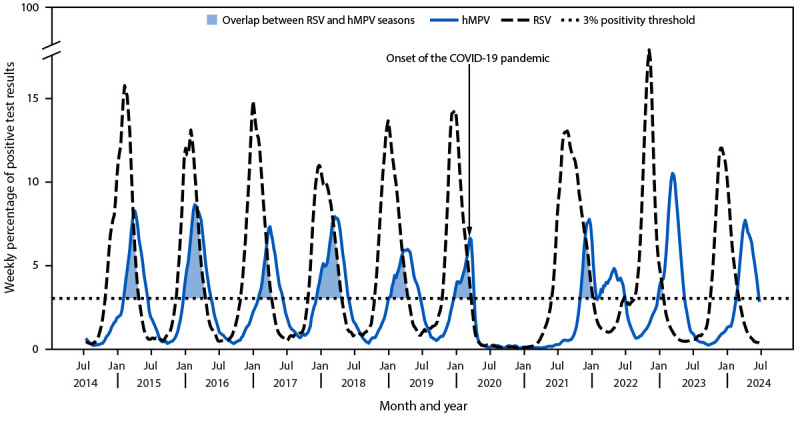
Weekly percentage of positive test results* for respiratory syncytial virus and human metapneumovirus — National Respiratory and Enteric Virus Surveillance System, United States, July 2014–June 2024^†^ **Abbreviations**: hMPV = human metapneumovirus; RSV = respiratory syncytial virus. * Data were smoothed using a 3-week, centered moving average. ^†^ The overlap between RSV and hMPV seasons represents the period before the RSV offset week (≥3% positive test results in the last of ≥2 consecutive weeks) and after the hMPV onset week (≥3% positive test results in the first of ≥2 consecutive weeks), nationally.

The prepandemic median hMPV season onset, peak, and offset occurred in early January, late March, and early June, respectively (Table 2), and the median season duration was 21 weeks (range = 15–25 weeks). The 2021–22 hMPV season was atypical, with geographically variable peaks.^§§^ As a result, the duration of the 2021–22 season (35 weeks) was longer than that of other seasons. In 2022–23 and 2023–24, hMPV seasonality shifted to more typical circulation patterns, similar to prepandemic seasons with peaks in March and April, respectively.

**TABLE 2:** Respiratory syncytial virus and human metapneumovirus season onset, peak, offset, and duration, by surveillance year, before, during, and after the COVID-19 pandemic — National Respiratory and Enteric Virus Surveillance System, United States, 2014–2024*

NREVSS yr	Onset mo (surveillance wk)^†^		Peak mo (surveillance wk)^†^		Offset mo (surveillance wk)^†^		Season duration, wks	
	RSV	hMPV	RSV	hMPV	RSV	hMPV	RSV	hMPV
**Preؘ–COVID-19 pandemic**								
2014–2015	Oct (43)	Feb (5)	Feb (6)	Apr (14)	Apr (16)	Jun (22)	27	18
2015–2016	Nov (45)	Dec (51)	Feb (5)	Feb (7)	Apr (15)	May (20)	23	22
2016–2017	Oct (42)	Jan (4)	Dec (52)	Mar (12)	Apr (15)	Jun (23)	26	20
2017–2018	Oct (42)	Dec (49)	Dec (51)	Mar (10)	Apr (16)	May (21)	27	25
2018–2019	Oct (41)	Jan (1)	Dec (51)	Apr (16)	Apr (16)	Jun (24)	28	24
2019–2020	Oct (42)	Dec (52)	Dec (51)	Mar (12)	Mar (12)	Apr (14)	23	15
**Six-season median**	**Oct (42)**	**Jan (53)**	**Dec (52)**	**Mar (12)**	**Apr (16)**	**Jun (22)**	**26.5**	**21**
**COVID-19 pandemic** ^§^								
2021–2022	May (21)	Oct (43)	Jul (30)	Dec (50)	Jan (1)	Jun (25)	33	35^¶^
2022–2023	Jun (24)	Dec (52)	Nov (44)	Mar (11)	Jan (3)	May (18)	32	19
Post–COVID-19 pandemic**								
2023–2024	Oct (40)	Feb (8)	Nov (47)	Apr (14)	Mar (9)	Jun (24)	22	17

**Abbreviations**: hMPV = human metapneumovirus; NREVSS = National Respiratory and Enteric Virus Surveillance System; RSV = respiratory syncytial virus.

* Using crude (unsmoothed) data, hMPV and RSV season onsets and offsets were defined as the first and last of ≥2 consecutive weeks, respectively, with ≥3% positive test results; the season peak was the week with the highest percentage of positive test results.

^†^ MMWR surveillance weeks can be found online. https://ndc.services.cdc.gov/wp-content/uploads/2021/02/MMWR_Week_overview.pdf

^§^ The 2020–21 hMPV and RSV seasons were excluded because of disruptions in virus circulation associated with the COVID-19 pandemic.

^¶^ During 2021–22, an atypical hMPV season occurred with peaks in early December (weeks 48 and 49) in U.S. Department of Health and Human Services Regions 4 and 6, early January (week 52) in Region 7, early March (week 10) in Region 9, late March (weeks 11 and 12) in Regions 5 and 8, late April (week 17) in Region 3, late May (week 21) in Region 1, and early June (week 22) in Regions 2 and 10. As a result, the duration of the 2021–22 season (35 weeks) was longer than other seasons. https://www.hhs.gov/about/agencies/iea/regional-offices/index.html

** The COVID-19 pandemic public health emergency declaration ended May 2023.

### RSV Circulation

Before the pandemic, the median onset, peak, and offset for RSV seasons occurred in late October, late December, and late April, respectively (Table 2). In the 2021–22 and 2022–23 seasons, RSV onset shifted to late May and mid-June, but the seasonal peaks during 2021–22 (late July) and 2022–23 (early November) differed considerably. The duration of both the 2021–22 and 2022–23 RSV seasons was longer (33 and 32 weeks, respectively) compared with prepandemic seasons (median = 26.5 weeks; range = 23–28 weeks). The 2023–24 RSV season onset occurred much later in October, but the November seasonal peak was similar to that during the 2022–23 season. In the two pandemic and one postpandemic seasons, RSV offsets occurred earlier in January or March compared with the prepandemic median offset in April.

### Overlap in RSV and hMPV Seasons

Before the pandemic, RSV and hMPV seasons overlapped considerably (Figure), with hMPV season onset occurring a median of 13.5 weeks before RSV offset (range = 11–19 weeks). During and after the pandemic, the median period of overlap in RSV and hMPV seasons was 3 weeks (range = 1–10 weeks). hMPV peak circulation consistently followed that of RSV by a median of 11.5 weeks before the pandemic (range = 2–17 weeks). During and after the pandemic, peak hMPV circulation followed RSV by a median of 19 weeks (range = 19–20 weeks), representing an additional 7.5 weeks between peaks.

RSV and hMPV season overlap varied regionally (Supplementary Figure, https://stacks.cdc.gov/view/cdc/177080#tabs-3). Before the pandemic, the median overlap ranged from 9–13 weeks (HHS Regions 1, 2, 3, 4, 5, 9, and 10) to 14–19 weeks (Regions 6, 7, and 8). During the 2021–22 season, periods of overlap varied from 0 weeks (Regions 2 and 10) and 1–4 weeks (Regions 5 and 9) to 5–8 weeks (Regions 1, 3, 4, 7, and 8) and 12 weeks (Region 6). In the 2022–23 and 2023–24 seasons, an overlap of 0–4 weeks was most common in all regions, except Region 6 (12 weeks) and Region 9 (6 weeks) during the 2022–23 season.

## Discussion

Before the COVID-19 pandemic, co-circulation of hMPV and RSV occurred during the winter months. However, seasonality for respiratory viruses was disrupted during the pandemic (*5*,*6*). RSV season offsets in early winter persisted through 2023–24, resulting in minimal overlap in hMPV and RSV circulation and an additional 7.5 weeks between seasonal peaks during and after the pandemic. Because RSV seasonality is returning to typical patterns, a higher degree of co-circulation of RSV and hMPV in 2024–25 and future seasons is expected. As of the week ending March 22, 2025, the RSV (4.4% positive test results) and hMPV (5.5% positive test results) seasons have overlapped for 5 weeks nationally. During future periods of co-circulation, the combined incidence of these respiratory illnesses might be higher than they were during the COVID-19 pandemic, when RSV and hMPV circulated separately. A meta-analysis published in 2020 found that RSV and hMPV co-infections were associated with higher odds of admission to pediatric intensive care units (7).

hMPV and RSV, both members of the *Pneumoviridae* family, cause acute respiratory illnesses and severe disease similarly in older adults and immunocompromised patients. However, risk groups and clinical management are different in pediatric populations. Children hospitalized with hMPV are significantly older than those hospitalized with RSV (*8**,**9*); unlike for hMPV, very young infants are at highest risk for severe RSV-associated disease. Overall, diagnoses of bronchiolitis and requirement of high-flow respiratory support are more frequent in children with RSV, whereas pneumonia diagnoses, and mechanical ventilation requirements are more frequent in children with hMPV (New Vaccine Surveillance Network, unpublished data, 2025).

### Limitations

The findings in this report are subject to at least three limitations. First, the threshold of 3% positive test results is an imprecise measure of seasonality, particularly at the local level, where the timing of circulation varies (*10*). Second, NREVSS is a passive and voluntary surveillance system; therefore, participating laboratories vary from season to season and might not be representative of all geographic areas (*4*). More laboratories reporting during and after the pandemic and increasing numbers of tests contributed to a larger number of hMPV detections and a higher percentage of positive results over time. These changes likely represent the combined effects of increased testing capacity and broader use of multipathogen testing panels driven by COVID-19 testing. This expanded ability to identify hMPV might have led to the detection of cases that would have otherwise remained etiologically undiagnosed in previous years. Thresholds for seasonality could also be affected by increased testing. Finally, patient demographic data are not collected in NREVSS, precluding analysis of hMPV circulation patterns across different population groups (e.g., proportions of tests performed for children and adults are unknown).

### Implications for Public Health Practice

Near real-time surveillance data are vital for monitoring hMPV, RSV, and other causes of seasonal surges in respiratory illness. Understanding hMPV seasonality and the timing of hMPV and RSV co-circulation, especially that co-circulation has declined since the COVID-19 pandemic, can guide the timing and prioritization of clinician-directed testing, supportive care needs, and health care system preparedness. Currently, no licensed antiviral therapies or vaccines are available for hMPV, whereas licensed RSV vaccines and monoclonal antibody products are expected to reduce the incidence of severe RSV disease in infants and older adults in the coming years. Future incidence studies could estimate the effect of prevention products during periods of co-circulation.

## Footnotes

*
https://ictv.global/report/chapter/pneumoviridae/pneumoviridae

†
https://www.cdc.gov/nrevss/php/dashboard/index.html

§ For RSV, the typical winter epidemic was absent in 2020–21, and the 2021–22 epidemic began in the spring; therefore, the surveillance years for 2021–22 and 2022–23 (COVID-19 pandemic seasons) were defined from early March (week 9) to late February (week 8) of the following year.

¶ The weekly percentage of positive test results was calculated by dividing the total number of positive test results by the total number of tests performed each week, multiplied by 100.

**
https://www.hhs.gov/about/agencies/iea/regional-offices/index.html

†† 45 C.F.R. part 46.102(l)(2), 21 C.F.R. part 56; 42 U.S.C. Sect. 241(d); 5 U.S.C. Sect. 552a; 44 U.S.C. Sect. 3501 et seq.

§§ Peaks occurred in early December (weeks 48 and 49) in HHS Regions 4 and 6, early January (week 52) in Region 7, early March (week 10) in Region 9, late March (weeks 11 and 12) in Regions 5 and 8, late April (week 17) in Region 3, late May (week 21) in Region 1, and early June (week 22) in Regions 2 and 10.
